# Does the Supplemental Nutrition Assistance Program Affect Hospital Utilization Among Older Adults? The Case of Maryland

**DOI:** 10.1089/pop.2017.0055

**Published:** 2018-04-01

**Authors:** Laura J. Samuel, Sarah L. Szanton, Rachel Cahill, Jennifer L. Wolff, Pinchuan Ong, Ginger Zielinskie, Charles Betley

**Affiliations:** ^1^ Department of Acute and Chronic Care, Johns Hopkins School of Nursing, Baltimore, Maryland.; ^2^ Department of Community-Public Health, Johns Hopkins School of Nursing, Baltimore, Maryland.; ^3^ Benefits Data Trust, Philadelphia, Pennsylvania.; ^4^ Department of Health Policy and Management, Johns Hopkins Bloomberg School of Public Health, Baltimore, Maryland.; ^5^ Northwestern University, Department of Economics, Northwestern University, Evanston, Illinois.; ^6^ Benefits Data Trust, Philadelphia, Pennsylvania.; ^7^ The Hilltop Institute, University of Maryland Baltimore County, Baltimore, Maryland.

**Keywords:** food assistance, health care utilization, hospitalization, older adults, socioeconomic status

## Abstract

This study sought to examine whether Supplemental Nutrition Assistance Program (SNAP) participation and benefit levels are associated with reduced subsequent hospital and emergency department utilization in low-income older adults. Study participants were 68,956 Maryland residents aged ≥65 years who were dually enrolled in Medicare and Medicaid (2009–2012). Annual inpatient hospital days and costs and emergency department visits were modeled as a function of either 1-year lagged SNAP participation or lagged SNAP benefit amounts, controlling for sociodemographic characteristics, autoregressive effects, year, health status, and Medicaid participation. SNAP participation (adjusted odds ratio [aOR] = 0.96, 95% confidence interval [CI]: 0.93, 0.99), and, among participants, each $10 increase in monthly benefits (aOR = 0.99, 95% CI: 0.99–0.99) are associated with a reduced likelihood of hospitalization, but not emergency department use. The authors estimate that enrolling the 47% of the 2012 population who were eligible nonparticipants in SNAP could have been associated with $19 million in hospital cost savings. Accounting for the strong effects of health care access, this study finds that SNAP is associated with reduced hospitalization in dually eligible older adults. Policies to increase SNAP participation and benefit amounts in eligible older adults may reduce hospitalizations and health care costs for older dual eligible adults living in the community.

## Introduction

One third of US older adults, comprising 13 million older adults, currently live on incomes less than 200% of the poverty level, according to Census data. It has long been known that adults living under or near the poverty line rely more heavily on emergency department (ED)-based health care and are hospitalized more often than their higher income peers.^1,2^ Excess hospital utilization in this population was once believed to be preventable by improving health care access. However, disparities exist among older adults who have health insurance through Medicare,^1^ and are not attributable to access to primary care providers.^3,4^ One study found that non–health sector resources are associated with a reduced risk of hospital readmission in low-income older adults,^5^ suggesting that social determinants of health affect hospital utilization.

Social service programs exist to help low-income seniors meet their basic needs. Specifically, food assistance programs, such as the Supplemental Nutrition Assistance Program (SNAP), provide supplemental household income for food, and may therefore improve health outcomes for lower income older adults. SNAP provided, on average, $129 in supplemental monthly income for an average of 1.3 people in an older adult household in 2014.^6^ This transfer comprises a relatively large supplemental income source for these adults, whose average monthly gross income was $876.^6^ Also, by targeting financial support toward food needs, SNAP can improve access to a higher quality diet for food insecure adults.^7^ There is evidence of reduced caloric intake, poorer dietary quality,^8^ and greater risk of hypoglycemia^9^ for low-income adults at the end of the month when funds run low. Therefore, greater SNAP benefits may facilitate chronic disease management for nutrition-sensitive conditions, which may account for evidence of reduced chronic disease hospital utilization.^10^ Also, because adults with food insecurity also often report cost-related medication nonadherence and difficulty paying bills,^11,12^ food assistance may allow them to use their limited money for medications and other health-related necessities.

Several studies have tested whether SNAP is associated with reduced hospital utilization, but results are inconsistent. For example, Medicaid inpatient hospital spending growth declined in Massachusetts following an increase in SNAP benefit amounts in that state^10^ and results were most dramatic among those with nutrition-sensitive chronic conditions, suggesting that improved chronic disease management contributed to the decline. However, in a study of older patients with diabetes that adjusted for self-management behaviors, SNAP participants had similar Medicare spending and a 7% greater risk of hospitalization than their peers.^13^ These results contradict the hypothesis that SNAP participation is associated with reduced hospitalization, but may be biased by adjusting for self-management, because SNAP's effect on hospitalization likely is partly related to improved disease management.

Understanding how SNAP relates to health outcomes of low-income older adults is particularly important and timely in light of recent interest in innovative approaches that target the social needs of older adults.^14^ Approximately 42% of income-eligible older adults participate in SNAP.^15^ Although expanding access to SNAP and increasing SNAP benefits would undoubtedly increase benefit expenditures, program costs are modest relative to hospital and emergency care costs. Understanding how SNAP participation and benefit amounts affect hospital utilization could inform policy directed at improving the value and impact of federal entitlement programs. Therefore, this study sought to quantify SNAP participation rates in a population of low-income older Maryland residents dually eligible for Medicare and Medicaid and test 2 hypotheses in this population. First, the study team hypothesized that SNAP participation is associated with reduced subsequent hospital utilization. Second, the team hypothesized that greater SNAP benefits are associated with reduced hospital utilization among participants. Dually eligible older adults are all likely income eligible for SNAP and have claims data suitable for these analyses.

## Methods

The sample for this study was comprised of the population of Maryland residents aged 65 years and older who were dually enrolled in Medicare and Medicaid at any time between 2010 and 2012. Because Medicaid income eligibility criteria were more stringent than those for SNAP, all were likely income eligible for SNAP. Study inclusion was determined for each calendar year. Hospital utilization was estimated as a function of prior year's SNAP participation and benefit amount. Nursing home residents are not eligible for SNAP. Therefore, individuals were excluded who resided in a nursing home for 9 months or more of the prior year, because they likely would not have had sufficient opportunity to enroll in SNAP during the prior calendar year. Individuals who were enrolled in fee-for-service Medicare and enrolled in Medicaid for at least 1 month during the calendar year were included for that year. Individuals who enrolled in Medicare or died during the calendar year were excluded if they had less than 6 months of claims data for the year, as in prior work.^16^ Medicare Advantage enrollees were excluded because full claims data for hospital ascertainment were not available.

Medicaid claims and sociodemographic data were merged with Medicare claims and SNAP program utilization data. The study was approved by the Institutional Review Boards at Johns Hopkins Medicine and University of Maryland, Baltimore County. A crosswalk between Medicare and Medicaid recipients' IDs generated for research purposes enabled data merging. The dependent variables in this study included annual inpatient hospital day count, annual inpatient hospital cost, and annual ED visit count, including visits that resulted in an inpatient admission and those resolved on an outpatient basis. The dependent variables were measured during each year, 2010–2012, while SNAP variables were measured from 2009–2011 to capture lagged effects. It was not possible to measure ED costs separately from inpatient hospital cost for visits resulting in admission. Inpatient hospital cost was measured by the total payment amount in Medicare claims data because Medicare is the primary payer of hospital care for dually eligible adults.

SNAP participants were compared to nonparticipants. Because individuals self-select into SNAP and may qualitatively differ from nonparticipants, the study team also measured the average benefit amount among participants (scaled in $10 increments) to evaluate potential dose response. Both SNAP variables were treated as time variant and modeled with 1-year lags, meaning that outcomes were regressed on the prior year's SNAP values.^17^ Lagged models minimize the threat of reverse causality, whereby individuals in poorer health select into SNAP participation. Sociodemographic variables included sex, race/ethnicity (analyzed as black, white, Hispanic, other and unknown [ref]), age, annual household income (in $1000 increments), and dummy variables indicating partially Medicaid eligible (ie, eligible for assistance with Medicare premiums and cost sharing, but not for full Medicaid coverage) and Medicaid eligibility based on medically needy spend down, both of which were considered to be socioeconomic proxies. Because poor health may contribute to both SNAP participation and hospital utilization, the study team measured the number of chronic conditions defined by a modified version of the Chronic Conditions Warehouse algorithm,^18^ based on diagnosis codes in either Medicare and Medicaid claims. Receipt of the Medicaid Home and Community-Based Services Waiver, which indicates functional limitations, was measured as a dichotomous variable. Finally, because older adults who participate in SNAP also may be more continuously enrolled in Medicaid, and because more continuous Medicaid participation is strongly associated with hospital utilization in dually eligible older adults,^5,19^ the study team measured the proportion of the year enrolled in Medicaid. Covariate data, except for chronic condition values, were drawn exclusively from Medicaid data, and all covariates were treated as time variant, except for sex and race/ethnicity.

### Statistical approach

SNAP participation and benefit amounts were modeled with 1-year lags in separate models. Zero-inflated negative binomial regression models estimated inpatient days and ED visits. The zero-inflated negative binomial fit the skewed distribution of inpatient hospital days and ED visits best because of the large frequency of nonusers during the year. Correlated outcomes were addressed by adjusting for autoregressive effects and applying robust standard error estimates.^17^ Time trends were addressed by adjusting for study year. Model 1 adjusted for sex, race/ethnicity, age, annual income, partially Medicaid eligible, Medicaid spend down eligibility, study year, autoregressive effects, chronic condition count, and Medicaid community waiver status. Model 2 additionally adjusted for proportion of the year participating in Medicaid. The inpatient hospital cost models are similar in concept, using a Heckman 2-step selection model for the same reason as the zero-inflated negative binomial model: there is a preponderance of zero utilization in any given year and a skewed distribution of spending. The Heckman model uses a probit specification in the first step to indicate a propensity for zero versus nonzero spending, and the second step is a weighted ordinary least squares specification of the amount of spending, conditioned on the propensity to be selected into the group of persons having hospital costs. Inpatient hospital cost models adjusted for all covariates.

Results from the Heckman 2-stage model also were used to estimate the potential cost implications of expanding access to SNAP to nonparticipants in 2012. Results from the first stage were used to estimate potential savings attributable to fewer hospital admissions and results from the second stage were used to estimate the potential savings attributable to less costly stays if admitted. The fully adjusted results from the first stage of the Heckman model provide the predicted difference in probability of hospital admission between SNAP participants and nonparticipants, assuming mean values for all model covariates. This value was then multiplied by the number of SNAP nonparticipants and the average cost of inpatient hospitalization to estimate the potential cost savings attributable to averted hospital admissions. The fully adjusted results from the second stage of the Heckman model provide the estimated percent reduction in cost among those admitted. This was multiplied by the expected number of hospitalizations in SNAP nonparticipants to calculate the potential cost savings attributable to less costly stays. The total potential cost savings is the sum of the cost results from both stages.

## Results

A total of 68,956 older adults in Maryland were dually enrolled in both fee-for-service Medicare and Medicaid and were eligible for this study at some point during 2010–2012. Of those, 53,646 individuals were eligible for the study in 2012, the most recent year of data. In 2012, 26% of participants were hospitalized and approximately 53% were enrolled in SNAP (Table 1). SNAP participants had a 3 percentage point lower likelihood than SNAP nonparticipants of being hospitalized, a 1 percentage point lower likelihood of having an ED visit, and were more likely to be younger, female, and black, Hispanic, or other race compared with being white or having unknown race/ethnicity. They were less likely to be partially eligible for Medicaid. Rates for hospitalization and the sociodemographic profile of the population remained mostly consistent across observation years (see online Supplementary Table S1; Supplementary Data are available online at www.liebertpub.com/pop). The average cost for inpatient admissions for participants hospitalized in 2012 was $25,091.

**Table 1.** Characteristics of Maryland Adults Aged ≥65 Dually Enrolled in Both Medicare and Medicaid, by Supplemental Nutrition Assistance Program Participation in 2012 (n = 53,646)

	*Total sample*	*SNAP participants (%)* 28,628 (53)	*Nonparticipants (%)* 25,018 (47)	P^a^
Age				
65–69 years	14,672 (27)	7305 (29)	7367 (26)	<0.01
70–74 years	11,621 (22)	4406 (18)	7215 (25)	
75–79 years	9976 (19)	4164 (17)	5812 (20)	
80–84 years	8098 (15)	3693 (15)	4405 (15)	
≥85 years	9279 (17)	5450 (22)	3829 (13)	
Sex				
Female	37,138 (69)	19,955 (70)	17,183 (69)	0.01
Male	16,508 (31)	8673 (30)	7835 (31)	
Race/Ethnicity				
Black	17,704 (33)	10,191 (36)	7513 (30)	<0.01
White	21,034 (39)	10,560 (37)	10,474 (42)	
Hispanic	2869 (5)	1694 (6)	1175 (5)	
Other	6835 (13)	4281 (15)	2554 (10)	
Unknown	5204 (10)	1902 (7)	3302 (13)	
Medicaid community waiver				
No	46,732 (87)	24,903 (87)	21,829 (87)	0.36
Yes	6914 (13)	3725 (13)	3189 (13)	
Partially Medicaid eligibile				
No	31,347 (58)	16,984 (59)	14,363 (57)	<0.01
Yes	22,299 (42)	11,644 (41)	10,655 (43)	
Medicaid eligible by spend down				
No	52,723 (98)	28,207 (99)	24,516 (98)	<0.01
Yes	923 (2)	421 (1)	502 (2)	
Mean number of chronic conditions	2.8	2.6	2.9	<0.01
Admitted to hospital				
No	40,031 (74)	21,238 (76)	18,793 (73)	<0.01
Yes	13,775 (26)	6734 (24)	7041 (27)	
Had emergency department visit				
No	31,674 (59)	16,634 (59)	15,040 (58)	0.03
Yes	22,132 (41)	11,338 (41)	10,794 (42)	

Limited to individuals who were enrolled in fee-for-service Medicare for ≥6 months of the year and who were not residing in a nursing home for more than 9 months of 2011.

^a^ Based on chi-square test statistic.

SNAP, Supplemental Nutrition Assistance Program.

Adjusting for sociodemographic and health characteristics, (Table 2, Model 1), SNAP participants had, on average, 14% lower odds of hospitalization and 10% lower odds of an ED visit in the subsequent year than nonparticipants. These associations were attenuated after additionally adjusting for Medicaid participation, but SNAP participation continued to be statistically significantly associated with 4% reduced odds of hospitalization in the final model (Model 2). Likewise, in models that adjusted for sociodemographic and health characteristics, SNAP participants had a 10% lower likelihood for each additional inpatient day if hospitalized and a 4% lower likelihood of each additional ED visit if they utilized it (Model 1), but SNAP participation was not associated with either outcome after additional adjustment for Medicaid participation. Adjusting for sociodemographic and health characteristics in SNAP participants, a $10 increase in monthly benefit amount was associated with 2% lower odds of either hospitalization or ED utilization (Model 1). In models that additionally adjusted for Medicaid participation, a $10 increase in monthly SNAP benefit continued to be statistically significantly associated with 1% reduced odds of hospitalization. Full model results are reported in Supplementary Tables S2 and S3.

**Table 2.** Associations Between Supplemental Nutrition Assistance Program Participation (*n* = 68,956) and Benefit Amount (n = 26,874) with Hospitalization and Emergency Department Visits, Maryland Adults Aged ≥65 Years Enrolled in Both Medicare and Medicaid (2010–2012)

	*Model 1*	*Model 2*^a^	*Model 1*	*Model 2*^a^
	*Any hospitalization*		*Any emergency department visits*	
	*OR (95% CI)*	*OR (95% CI)*	*OR (95% CI)*	*OR (95% CI)*
Previous year SNAP participation (n = 68,956)	0.86 (0.84–0.89)	0.96 (0.93–0.99)	0.90 (0.83–0.97)	0.98 (0.91–1.06)
Previous year mean monthly SNAP amount in participants ($10) (n = 26,874)	0.98 (0.97–0.98)	0.99 (0.99–0.99)	0.98 (0.98–0.99)	0.99 (0.99–1.00)

	*Number of inpatient hospital days among the hospitalized*		*Number of emergency department visits among utilizers*	
	*IRR (95% CI)*	*IRR (95% CI)*	*IRR (95% CI)*	*IRR (95% CI)*
Previous year SNAP participation (n = 68,956)	0.90 (0.81–0.99)	0.92 (0.82–1.03)	0.96 (0.93–0.99)	0.98 (0.95–1.01)
Previous year mean monthly SNAP amount in participants ($10) (n = 26,874)	0.99 (0.98–0.99)	0.99 (0.99–1.00)	0.99 (0.99–1.00)	1.00 (1.00–1.00)

Associations estimated from zero-inflated negative binomial regression estimated with robust standard errors. All models adjusted for autoregressive effects, study year, age, sex, race/ethnicity, annual income, partial Medicaid eligibility, Medicaid spend down eligibility, chronic condition count, and Medicaid community waiver status.

^a^ Model additionally adjusted for proportion of year participating in Medicaid.

CI, confidence interval; IRR, incident rate ratio; OR, odds ratio; SNAP, Supplemental Nutrition Assistance Program.

In fully adjusted models, SNAP participants were 1.5 percentage points less likely to incur an inpatient hospital expense (ie, be hospitalized; Table 3). Among those who were hospitalized, SNAP participants had 5.8% lower expenses than nonparticipants. Therefore, the study team estimates that expanding SNAP benefits to the 25,018 nonparticipants in 2012 could have been associated with total savings of $19 million, with approximately half of the savings ($9.4 million) related to an estimated 375 averted admissions and the other half ($9.7 million) related to less costly hospital stays (Table 4). Among SNAP participants, a $10 increase in SNAP was associated with a 0.2 percentage point lower probability of incurring inpatient-related hospital costs and a 1% lower average inpatient cost for those who were hospitalized (Table 3).This savings is not included in the cost savings estimation to avoid double counting.

**Table 3.** Associations Between Supplemental Nutrition Assistance Program Participation (n = 68,956) and Benefit Amount (n = 26,874) with Inpatient Hospital Expenditures, Maryland Adults Aged ≥65 Years Enrolled in Both Medicare and Medicaid (2010–2012)

	*Any hospitalization*
	*Marginal change in probability for any >0 hospital cost (95% CI)*^a^
Previous year SNAP participation (n = 68,956)	−1.5% (−2.0% to −1.1%)
Previous year mean monthly SNAP amount in participants ($10) (n = 26,874)	−0.2% (−0.2% to −0.1%)

	*Inpatient hospital Medicare cost among the hospitalized*
	*Estimated elasticity for ln(cost) (95% CI)*
Previous year SNAP participation (n = 68,956)	−5.8% (−8.4% to −3.3%)
Previous year mean monthly SNAP amount in participants ($10) (n = 26,874)	−1.0% (−1.3% to −0.7%)

Associations estimated from Heckman regression model, adjusted for autoregressive effects, study year, age, sex, race/ethnicity, annual income, partial Medicaid eligibility, Medicaid spend-down eligibility, chronic condition count, Medicaid community waiver status, and proportion of year participating in Medicaid.

^a^ Evaluated at means of all covariates.

CI, confidence interval; SNAP, Supplemental Nutrition Assistance Program.

**Table 4.** Steps to Obtain Cost Savings of Expanding the Supplemental Nutrition Assistance Program to Nonparticipants in 2012 (n = 25,018), Based on Heckman Model Estimates

*Cost savings from averted admissions*	
Change in probability of any admission in the year (%)^a^	1.5
Multiplied by: Number of nonparticipants^b^	25,018
Gives: Estimated number of averted admissions	375
Multiplied by: Average annual cost of inpatient admissions ($)^c^	25,091
Gives: Estimated cost savings from averted admissions ($)	9,415,900
*Cost savings from less costly hospital stays*	
Number of nonparticipants admitted to hospital^b^	7041
Less: Estimated number of averted admissions	375
Gives: Estimated no. of nonparticipants still admitted to hospital	6666
Percentage change in cost for admitted persons (%)^a^	5.8
Multiplied by: Average annual cost of inpatient admissions ($)^c^	25,091
Gives: Reduction in average cost for admitted persons ($)	1455
Multiplied by: Estimated no. of nonparticipants still admitted to hospital	6666
Gives: Estimated cost savings from less costly hospital stays ($)	9,700,490
*Total cost savings*	
Total estimated cost savings ($)	19,116,390

^a^ See Table 3.

^b^ See Table 1.

^c^ Estimated based on participants who were hospitalized in 2012.

## Discussion

Results from this study indicate that SNAP participation and increased SNAP benefits among participants were associated with reduced hospitalization rates, but not ED visit rates, in dually eligible older adults. Notably, in this study of older adults who should be eligible, only about half participated in SNAP. Despite the vast body of literature documenting poor health in low-income older adults, effective strategies to improve health outcomes for this vulnerable group remain scant. In this study, SNAP benefits were related to lower hospital utilization in a population that was continuously enrolled in Medicare and in regression models that adjust for the proportion of the year enrolled in Medicaid. It is notable that all had access to both Medicare and Medicaid because policy makers have increased access to health care for low-income groups thinking that that alone would reduce high hospital utilization in low-income groups. Indeed, SNAP associations in this study were attenuated after adjusting for the proportion of the year with Medicaid coverage, and evidence elsewhere shows that continuous Medicaid coverage predicts less hospital-based care.^19^ However, results from the present study add to evidence^1,3–5^ suggesting that high hospital utilization is attributable to both social and medical determinants of health. More specifically, these results identify food assistance as a social determinant of hospital utilization, but not ED utilization. Therefore, SNAP may predict fewer hospital admissions, even if it does not decrease the frequency of ED visits. Results from other studies suggest that improving health care access also may not reduce ED utilization.^20,21^ Together, these results suggest that hospital and ED utilization are attributable to different mechanisms. Importantly, inpatient hospital admission is determined by health care providers, whereas visiting the ED is generally the decision of the individual. Therefore, inpatient hospital utilization is likely a better measure of individual health status than ED utilization.

These findings supplement evidence that investment in SNAP may improve health outcomes and reduce inpatient hospital spending. For example, the American Recovery and Reinvestment Act of 2009, which expanded SNAP eligibility and increased the benefit amount, has been credited with reducing the prevalence of food insecurity in SNAP income-eligible US households by 2%^22^ and may have slowed growth in Medicaid inpatient hospital spending.^10^ These results build on prior studies by demonstrating that not only SNAP participation, but greater benefit amounts among participants, is associated with reduced hospitalization rates. This is notable because participation is susceptible to self-selection, but benefit amounts are assigned based largely on household financial need. Therefore, benefit amount results are less susceptible to confounding and reverse causality than participation results.

In this study, SNAP participation also was associated with less costly stays among those who were hospitalized. Based on the models, the study team estimates that expanding SNAP access to nonparticipating dual eligible older adults in Maryland could have resulted in inpatient hospital cost savings of $19 million in 2012. Based on the average per capita costs of the SNAP program, the team estimates that the federal government would spend approximately $39 million if they extended SNAP benefits to the 2012 income-eligible nonparticipants in the study sample. Therefore, ignoring issues of inefficiencies in taxation, approximately half of the cost of administering SNAP could be recouped by the federal government in reduced Medicare inpatient hospital spending. Further work is needed to quantify the potential savings attributable to changes in other health care utilization outcomes.

There are at least 2 potential reasons for the associations found in this study. First, SNAP participation reduces food insecurity^23,24^ and minimizes the adverse effect of food insecurity on dietary quality and obesity.^7^ This is noteworthy because food insecurity is linked to worse dietary quality,^8^ increased risk of chronic diseases,^25^ and increased risk of hospitalization.^26^ Therefore, it is plausible that improved access to SNAP and increased benefits for participants may improve food security and dietary quality in households of low-income older adults, and this may reduce hospital utilization.

An alternative potential reason for these results is that SNAP provides supplemental income that reduces financial strain for older adults living under or near the poverty line. Financial strain, or the lack of adequate money for basic needs, is linked with earlier mortality^27^ and greater risk for both malnutrition^28^ and disability,^29^ which contribute to hospitalization.^5,30^ Individuals experiencing financial strain struggle to afford food, heat, and health care,^11,12^ and may be forced to choose between these basic needs. Therefore, improving access to programs meeting any basic need will improve an individual's ability to afford the other basic needs. This idea is consistent with evidence of increased access to food after implementation of Medicare Part D, which improved financial access to medications for older adults.^31^ Therefore, it is also plausible that any supplemental income could be associated with reduced subsequent hospital-based care for low-income older adults because it enhances their ability to meet basic needs and reduces finance-related stress exposure.

### Limitations and strengths

As with other SNAP studies, SNAP participants may differ from nonparticipants on unmeasured characteristics. This may have biased associations and the study cost savings calculations. For example, older adults who do not receive SNAP may benefit from other meal assistance programs, such as Meals on Wheels or congregate meals. This likely would bias associations toward the null because of unmeasured receipt of food assistance in the control group. Conversely, older adults who participate in SNAP may be more likely to enroll in other public benefits programs, which could confound results if such programs collectively reduce hospital utilization. Also, this study is limited to an older adult population, precluding measurement of the cumulative lifetime participation in food programs, and making results susceptible to survival bias. Conversely, however, these results suggest that greater SNAP benefits may confer a health benefit for low-income adults, even at older ages. In addition, lag times for potential health effects of food assistance programs are unknown. Lagged effects may be longer than the 1 year modeled in this study, so the associations between SNAP and hospital utilization may be underestimated. Conversely, the study team could have missed key proximal effects by lagging the models for a year. Also, averted hospital stays may differ in cost from the typical hospital stay, which would bias the cost savings estimate. This study is strengthened by using data for an entire state population of dually eligible older adults, reducing nonresponse bias in a low-income group. Also, this study is less susceptible to reverse causality than previous cross-sectional studies because of use of lagged exposure modeling.

## Public Health Implications

This study found low rates of SNAP program participation (only 53% participated in the most recent study year) in the population of dually eligible Maryland older adults, all of whom are likely income eligible for SNAP. National data estimates a participation rate of 42% among income eligible older adults,^15^ suggesting that the program is underutilized. Results from this study suggest that improved access to SNAP may reduce hospitalization for low-income older adults. Together, these results suggest that strategies to improve access to SNAP for eligible older adults likely will improve health outcomes, despite years of accumulated exposure to food insecurity and financial strain in this population. These results have implications for practice and policy.

### Practice implications

Efforts to increase SNAP participation may include targeted eligibility screening and enrollment assistance. Older adults tend to underutilize the program.^15^ Health care providers and health care payers can invest in efforts to screen for food insecurity and income eligibility for SNAP and facilitate SNAP enrollment.^32^ Resources are available to support enrollment and advocacy efforts nationally and state-level advocacy organizations. Community health workers, primary care provider practices, and not-for-profit service providers are well positioned to provide such screening and referral, but they need to be compensated and supported for services to address social and economic determinants of health.

### Policy implications

Policy actions can improve access to SNAP and increase benefit amounts. Several specific policy strategies can facilitate the SNAP enrollment process for older adults. First, states can reduce enrollment requirements by implementing the Elderly Simplified Application Project. This program, implemented in 7 states,^33^ streamlines income and expense verification by matching data from existing sources, extends certification periods to 36 months, and waives the recertification interview.^34^ Second, states can coordinate SNAP and Supplemental Security Income enrollment, as is done in the Combined Application Project.^35^ This program increased SNAP participation rates in South Carolina at a time when national rates were declining^35^ and is currently implemented in 18 states.^33^ Third, states can leverage administrative data from Medicaid, the Low Income Home Energy Assistance Program (LIHEAP), and SNAP programs, as Maryland has, to identify income eligible older adults who are not enrolled in SNAP and conduct targeted outreach to increase participation.^36^ The Affordable Care Act incentivizes states to harmonize administrative data sets to facilitate Medicaid enrollment, and these efforts can facilitate SNAP enrollment as well.

Besides enhancing beneficiary access, states can enhance benefit amounts for vulnerable older adults. For example, the State of Maryland recently passed legislation ensuring that all SNAP beneficiaries aged 62 and older receive a minimum benefit of $30 monthly by supplementing the federal benefit with state funds as needed. Furthermore, efforts to significantly cut federal spending on SNAP benefits through block granting or other structural changes may have adverse consequences.

## Conclusion

This study found that SNAP participation and greater benefit amounts are associated with lower inpatient hospital utilization in a state population of low-income older adults. These findings have public health implications because the majority of US older adults who are income eligible for SNAP do not participate. As public and private sector health care partners shift to outcomes-driven, value-based care, social service programs such as SNAP will be a critical tool in improving health outcomes for low-income seniors across the country.

## Supplementary Material

Supplemental data

Supplemental data

Supplemental data
