# Supplementary material for: Does the Supplemental Nutrition Assistance Program Affect Hospital Utilization Among Older Adults? The Case of Maryland
Source: Popul Health Manag. 2018 Apr 1;21(2):88–95. doi: 10.1089/pop.2017.0055 (PMC5906726; doi:10.1089/pop.2017.0055)
Supplement: Supplemental data [file Supp_Table1.pdf]

Supplementary Data

Supplementary Table S1. Characteristics of Maryland Adults Aged ≥65 Dually Enrolled in Both Medicare and Medicaid, by Supplemental Nutrition Assistance Program Participation, 2010–2012

	2010 (n = *46,447*)			*2011* (n = *49,189*)			*2012* (n = *53,646*)		
	SNAP participants (%) 19,152 (41)	*SNAP nonparticipants (%) 27,295 (59)*	*Total*	*SNAP participants (%) 24,523 (50)*	*SNAP nonparticipants (%) 24,568 (50)*	*Total*	*SNAP participants (%) 28,628 (53)*	*SNAP nonparticipants (%) 25,018 (47)*	*Total*
Age			$P<0.01$			$P<0.01$			$P<0.01$
65–69	4976 (26)	6696 (25)	6088 (25)	6088 (32)	6943 (25)	13,031 (28)	7305 (29)	7367 (26)	14,672 (27)
70–74	4952 (26)	5387 (20)	6248 (22)	6248 (33)	4534 (17)	10,782 (23)	4406 (18)	7215 (25)	11,621 (22)
75–79	3827 (20)	5002 (18)	5001 (19)	5001 (26)	4316 (16)	9317 (20)	4164 (17)	5812 (20)	9976 (19)
80–84	2922 (15)	4496 (16)	3783 (16)	3783 (20)	3704 (14)	7487 (16)	3693 (15)	4405 (15)	8098 (15)
≥85	2475 (13)	5714 (21)	3403 (18)	3403 (18)	5071 (19)	8474 (18)	5450 (22)	3829 (13)	9279 (17)
Gender			$P<0.01$			$P<0.01$			$P=0.01$
Female	13,036 (68)	19,512 (71)	32,548 (70)	16,921 (88)	17,114 (63)	34,035 (73)	19,955 (70)	17,183 (69)	37,138 (69)
Male	6116 (32)	7783 (29)	13,899 (30)	7602 (40)	7454 (27)	15,056 (32)	8673 (30)	7835 (31)	16,508 (31)
Race/Ethnicity			$P<0.01$			$P<0.01$			$P<0.01$
Black	6098 (32)	9314 (34)	15,412 (33)	8453 (34)	7775 (32)	16,228 (33)	10,191 (36)	7513 (30)	17,704 (33)
Caucasian	7826 (41)	11,169 (41)	18,995 (41)	9163 (37)	10,351 (42)	19,514 (40)	10,560 (37)	10,474 (42)	21,034 (39)
Hispanic	1045 (5)	1330 (5)	2375 (5)	1399 (6)	1127 (5)	2526 (5)	1694 (6)	1175 (5)	2869 (5)
Other	3035 (16)	2916 (11)	5951 (13)	3762 (15)	2520 (10)	6282 (13)	4281 (15)	2554 (10)	6835 (13)
Unknown	1148 (6)	2566 (9)	3714 (8)	1746 (7)	2795 (11)	4541 (9)	1902 (7)	3302 (13)	5204 (10)
Medicaid community wavier			$P<0.01$			$P<0.01$			$P=0.36$
No	17,146 (90)	25,183 (92)	42,329 (91)	21,278 (87)	21,744 (89)	43,022 (88)	24,903 (87)	21,829 (87)	46,732 (87)
Yes	2006 (10)	2112 (8)	4118 (9)	3245 (13)	2824 (11)	6069 (12)	3725 (13)	3189 (13)	6914 (13)
Partially Medicaid eligible			$P=0.05$			$P<0.01$			$P<0.01$
No	11,582 (60)	16,150 (59)	27,732 (60)	15,360 (63)	12,833 (52)	28,193 (57)	16,984 (59)	14,363 (57)	31,347 (58)
Yes	7570 (40)	11,145 (41)	18,715 (40)	9163 (37)	11,735 (48)	20,898 (43)	11,644 (41)	10,655 (43)	22,299 (42)
Medicaid eligible by spenddown			$P=0.15$			$P<0.01$			$P<0.01$
No	18,839 (98)	26,895 (99)	45,734 (98)	24,181 (99)	24,070 (98)	48,251 (98)	28,207 (99)	24,516 (98)	52,723 (98)
Yes	313 (2)	400 (1)	713 (2)	342 (1)	498 (2)	840 (2)	421 (1)	502 (2)	923 (2)
Admitted to hospital			$P<0.01$			$P<0.01$			$P<0.01$
No	10,771 (75)	22,323 (70)	33,094 (71)	15,249 (75)	20,908 (72)	36,157 (74)	17,462 (76)	22,569 (73)	40,031 (74)
Yes	3680 (26)	9719 (30)	13,399 (29)	4979 (25)	8053 (28)	13,032 (27)	5439 (24)	8336 (27)	13,775 (26)
Had emergency department visit			$P<0.01$			$P<0.02$			$P=0.66$
No	8937 (62)	18,635 (58)	27,572 (59)	12,081 (60)	16,986 (59)	29,067 (59)	13,456 (59)	18,218 (59)	31,674 (59)
Yes	5514 (38)	13,407 (42)	18,921 (41)	8147 (40)	11,975 (41)	20,122 (41)	9445 (41)	12,687 (41)	22,132 (41)
Mean number of chronic conditions			$P<0.01$			$P<0.01$			$P<0.01$
	2.4	2.1	2.2	3.3	3	3.1	2.6	2.9	2.8

SNAP, Supplemental Nutrition Assistance Program.
