# Supplementary material for: Does the Supplemental Nutrition Assistance Program Affect Hospital Utilization Among Older Adults? The Case of Maryland
Source: Popul Health Manag. 2018 Apr 1;21(2):88–95. doi: 10.1089/pop.2017.0055 (PMC5906726; doi:10.1089/pop.2017.0055)
Supplement: Supplemental data [file Supp_Table3.pdf]

Supplementary Table S3. Associations Between Supplemental Nutrition Assistance Program Benefit Amount with Hospitalization and Emergency Department Visits, Maryland Adults Aged ≥65 Years Enrolled in Both Medicare and Medicaid (2010–2012)

	Any hospitalization OR (95% CI)	*Any emergency department visit OR (95% CI)*
Female	0.89 (0.85–0.93)	1.35 (1.21–1.52)
Age	1.03 (1.03–1.03)	1.03 (1.02–1.04)
Race (missing is ref.)		
Black	1.24 (1.14–1.35)	2.13 (1.71–2.65)
Caucasian	1.10 (1.01–1.20)	1.33 (1.08–1.64)
Hispanic	0.80 (0.71–0.90)	2.00 (1.39–2.88)
Other	0.63 (0.57–0.69)	1.04 (0.78–1.39)
Previous year mean monthly SNAP amount ($10)	0.99 (0.99–0.99)	0.99 (0.99–1.00)
Chronic conditions	1.44 (1.42–1.45)	1.37 (1.35–1.40)
Annual income ($1,000)	1.00 (1.00–1.00)	1.00 (1.00–1.01)
Medicaid eligible via spend-down	2.18 (1.84–2.58)	1.30 (0.76–2.21)
Partial Medicaid eligibility	0.69 (0.65–0.74)	0.92 (0.78–1.09)
Proportion of year covered by Medicaid	0.15 (0.14–0.17)	0.08 (0.03–0.18)
Previous year inpatient hospital days/ED visit count	1.08 (1.07–1.09)	2.77 (2.29–3.35)
Medicaid community waiver	0.83 (0.78–0.89)	1.07 (0.89–1.31)
2011 year dummy	0.62 (0.58–0.65)	0.39 (0.31–0.50)
2012 year dummy	0.66 (0.63–0.69)	0.44 (0.35–0.55)
	Number of inpatient hospital days among the hospitalized IRR (95% CI)	*Number of emergency department visits among utilizers IRR (95% CI)*
Female	0.89 (0.85–0.93)	0.84 (0.80–0.87)
Age	0.99 (0.99–0.99)	1.00 (1.00–1.00)
Race (missing is ref.)		
Black	0.97 (0.89–1.06)	1.19 (1.08–1.30)
Caucasian	0.90 (0.82–0.98)	1.07 (0.97–1.18)
Hispanic	0.78 (0.67–0.91)	0.73 (0.65–0.82)
Other	0.78 (0.69–0.87)	0.55 (0.49–0.61)
Previous year mean monthly SNAP amount ($10)	1.00 (0.99–1.00)	1.00 (1.00–1.00)
Chronic conditions	1.03 (1.02–1.03)	1.13 (1.12–1.14)
Annual income ($1,000)	1.02 (1.01–1.02)	1.00 (1.00–1.01)
Medicaid eligible via spend-down	0.92 (0.82–1.03)	1.35 (1.17–1.55)
Partial Medicaid eligibility	0.80 (0.76–0.84)	0.88 (0.83–0.92)
Proportion of year covered by Medicaid	0.47 (0.43–0.51)	0.77 (0.71–0.84)
Previous year inpatient hospital days/ED visit count	1.02 (1.02–1.02)	1.11 (1.10–1.12)
Medicaid community waiver	0.94 (0.89–1.00)	0.94 (0.88–1.00)
2011 year dummy	0.92 (0.87–0.97)	0.97 (0.93–1.02)
2012 year dummy	0.93 (0.88–0.98)	1.03 (0.98–1.08)

Associations estimated from zero-inflated negative binomial regression estimated with robust standard errors. All models adjusted for autoregressive effects, study year, age, sex, race/ethnicity, annual income, partial Medicaid eligibility, Medicaid spend-down eligibility, chronic condition count, Medicaid community waiver status and proportion of year participating in Medicaid.

CI, confidence interval; ED, emergency department; IRR, incident rate ratio; OR, odds ratio.
